# Sex workers as peer health advocates: community empowerment and transformative learning through a Canadian pilot program

**DOI:** 10.1186/s12939-017-0655-2

**Published:** 2017-08-30

**Authors:** Cecilia Benoit, Lynne Belle-Isle, Michaela Smith, Rachel Phillips, Leah Shumka, Chris Atchison, Mikael Jansson, Charlotte Loppie, Jackson Flagg

**Affiliations:** 1 Centre for Addictions Research of BC, University of Victoria, PO Box 1700 STN CSC, Victoria, BC V8W 2Y2 Canada; 2 Department of Sociology, University of Victoria, Victoria, Canada; 3 Canadian AIDS Society, Ottawa, Canada; 4 Peers Victoria Resource Society, Victoria, Canada; 5 Centre for Indigenous Research and Community-Led Engagement, University of Victoria, Victoria, Canada

**Keywords:** Canada, Community empowerment, Health inequities, Peer-led interventions, Sex workers, Stigma

## Abstract

**Background:**

Social marginalization and criminalization create health and safety risks for sex workers and reduce their access to health promotion and prevention services compared to the general population. Community empowerment-based interventions that prioritize the engagement of sex workers show promising results. Peer-to-peer interventions, wherein sex workers act as educators of their colleagues, managers, clients and romantic partners, foster community mobilization and critical consciousness among sex workers and equip them to exercise agency in their work and personal lives.

**Methods:**

A pilot peer health education program was developed and implemented, with and for sex workers in one urban centre in Canada. To explore how the training program contributed to community empowerment and transformative learning among participants, the authors conducted qualitative interviews, asked participants to keep personal journals and to fill out feedback forms after each session. Thematic analysis was conducted on these three data sources, with emerging themes identified, organized and presented in the findings.

**Results:**

Five themes emerged from the analysis. Our findings show that the pilot program led to reduced internalized stigma and increased self-esteem in participants. Participants’ critical consciousness increased concerning issues of diversity in cultural background, sexual orientation, work experiences and gender identity. Participants gained knowledge about how sex work stigma is enacted and perpetuated. They also became increasingly comfortable challenging negative judgments from others, including frontline service providers. Participants were encouraged to actively shape the training program, which fostered positive relationships and solidarity among them, as well as with colleagues in their social network and with the local sex worker organization housing the program. Resources were also mobilized within the sex worker community through skills building and knowledge acquisition.

**Conclusion:**

The peer education program proved successful in enhancing sex workers’ community empowerment in one urban setting by increasing their knowledge about health issues, sharing information about and building confidence in accessing services, and expanding capacity to disseminate this knowledge to others. This ‘proof of concept’ built the foundation for a long-term initiative in this setting and has promise for other jurisdictions wishing to adapt similar programs.

**Electronic supplementary material:**

The online version of this article (10.1186/s12939-017-0655-2) contains supplementary material, which is available to authorized users.

## Background

Social marginalization and a criminalised working environment create elevated health risks for sex workers and reduce their access to health promotion and prevention services compared to the general population [1, 2]. These risks vary greatly depending on social demographics, political structures and social policies (e.g., policies regulating sex work, stigma, discrimination, etc.), community engagement, including availability of sex work agencies and harm reduction community services, and workplace specific factors such as occupational health and safety and licensing regimes [3–6]. Sex workers who identify as Indigenous, men, trans, frequently use drugs or alcohol, and/or meet their clients outdoors have poorer health and greater unmet health care needs than others in the sex industry [7–9] and less access to health services [10–12].

Research examining the effectiveness of health promotion programs for sex workers highlights the importance of community empowerment-based responses that prioritize the engagement of members of the targeted population in the development and delivery of the programs [13, 14]. The Sonagachi Project in Kolkata, India is a case in point. Initiated in 1992 as a program to prevent sexually transmitted and bloodborne infection (STBBI) transmission among sex workers, it has since transitioned into a comprehensive health, safety and human rights program largely run by sex workers themselves [15]. The Pumwani Majengo project, in Nairobi, Kenya, also based on community empowerment and peer engagement principles, has faced political and financial constraints throughout its existence, but is supported by strong evidence that indicates its important role in preventing STBBIs among sex workers. Despite the difficult social context in which the project is embedded, it has since expanded to several other Kenyan cities [2].

Community empowerment, a concept often used in health promotion and peer education [16–20], has been defined as “a collective process through which the structural constraints to health, human rights and well-being are addressed by sex workers to create social and behavioural changes, and access to health services” (p. 19) [21]. Community empowerment interventions foster community mobilization and critical consciousness among sex workers to transform power relations [2] and enable sex workers to take control of their lives [22]. Community empowerment thus follows a dynamic continuum involving personal empowerment, the development of small mutual groups, community mobilization, and social and political action [21]. This conceptualisation of community empowerment draws on the emancipatory pedagogy of Paolo Freire and integrates dimensions of social influence and transforming power relations [2, 23]. In comparing two HIV prevention programs involving sex workers, Cornish and Campbell [24] note: The Indian project’s relative success was facilitated (1) by a more stable and supportive social, material and political context, and (2) by a community development ethos which devoted significant resources to sex workers’ involvement, ownership and empowerment, as opposed to a biomedical approach which marginalized sex workers’ concerns (p.123).

Successful community empowerment processes thus involve peer-to-peer interventions wherein sex workers act as authentic educators of their colleagues, managers, clients and romantic partners [2]. Peer education has been shown in some countries to be more effective than traditional health promotion approaches [22, 25, 26] because it is more successful in reaching both hidden and stigmatized populations that do not engage easily with service providers [4, 27–30]. Peer-led initiatives such as the Mobile Access Project in Vancouver, Canada aid in establishing supportive relationships that enable sex workers to access health and social resources [31, 32].

Finally, community empowerment interventions aim to affect societal change by striving to transcend oppressive conditions through a transformative learning process [2, 33]. This process takes place when people “meet in cooperation in order to transform the world.” (p. 167) [23]. Transformative learning occurs when people develop the critical consciousness to deconstruct prevailing ideologies, recognise the social, political, economic and personal constraints on their freedom, and gain awareness that they can be agents of change [33].

This paper presents findings from a pilot study aimed at increasing sex worker community empowerment and transformative learning for sex workers in an urban centre in Canada in order to enhance the effectiveness of health prevention and treatment services. Specifically, the pilot project focused on facilitating peer-based community leadership by designing and evaluating a small experimental STBBI prevention strategy led by sex workers as peer educators. We expect that the piloting of such a peer-based knowledge exchange initiative that targets sex workers’ personal and workplace networks will empower them to shape personal practices around health promotion and prevention strategies, and will improve their access to health and social services within the local community.

## Methods

### Study setting

Funded through a 1-year catalyst grant from the Canadian Institutes for Health Research, the project was initiated by the local peer-run sex worker agency in a middle-size Canadian city as an opportunity to increase community empowerment and transformative learning in order to enhance the effectiveness of STBBI treatment and prevention services for the local sex workers community that is diverse regarding demographic characteristics, health status and self-esteem [34]. This organization and the lead author have been involved in community-academic research initiatives for over 20 years. The size of the local sex work population is unknown but in any 1 year perhaps 1000 adults sell sexual services on a full or part-time basis. The project connected the sex worker organization with other community-based agencies, health service organizations and academic researchers to share knowledge related to sexual health promotion, prevention and protection. The research setting is well-known for its collaboration among municipal leaders, police and justice officials and health and social services providers to reduce marginalization of sex workers in the community. In recent years, despite punitive federal prostitution laws, the regional police, healthcare and social service providers and advocacy groups have worked cooperatively to improve the health and safety of sex workers and those with whom they interact in work and private life [35, 36]. These relations likely affect the perceptions of sex workers, and assessment of their options to deal with health challenges.

### Conceptual framework

In addition to serving as a theoretical foundation for our pilot study, community empowerment and transformative learning conceptual frameworks were useful for our data analysis, as explained below. Several frameworks have been offered to measure community empowerment (see [16] for a systematic review). Most useful for this qualitative exploration of community mobilization and structural change were conceptual discussions and frameworks that unpacked the concept of community empowerment and provided useful domains [16–18, 37–40].

We also drew upon Brookfield’s [41] learning theory that identifies seven learning tasks related to transformative learning: 1) Challenging ideologies: where people challenge values, beliefs, myths, explanations, and justifications embedded in language, social habits, and cultural forms; 2) Contesting hegemony: where people challenge the conditions that serve those in power; 3) Unmasking power: where people recognize how power is exercised in social interactions and relations; 4) Overcoming alienation: where people develop a sense of agency and are able to be themselves in an authentic way; 5) Learning liberation: where people learn to escape ideological domination; 6) Reclaiming reason: where people apply reason to examine how their lives our shaped by the lifeworld; and 7) Practicing democracy: where people use rational discourse, pay attention to ideal speech conditions, and pay attention to power structures related to diversity. The community empowerment frameworks and Brookfield’s leaning theory helped us to interpret our pilot findings presented below.

### Data collection

The training program reported in this article aimed at working with sex workers as peer educators to develop and pilot an innovative intervention targeted at their workplace as well as their intimate partnerships. Evidence related to the effectiveness of community empowerment interventions with sex workers was compiled to guide the development of the program and developed in consultation with the community partners on the research team. The program adopted a community-based participatory research approach guided by a research team comprising sex workers, representatives from service organizations and healthcare clinics for marginalised populations, health service managers and researchers. The pilot training program curriculum was flexible and adapted as the program was delivered, based on input from participants.

Purposive criterion sampling was used to recruit participants through a hiring process; criteria included interest in improving health and access to health care services for sex workers, being 19 years of age or older and currently engaged in sex work in the research city and having some demonstrated leadership skills and networking abilities. This criterion was deemed important as the participants were expected to perform their educator roles after a relatively short period of training. A hiring committee was established with members of the research team to recruit a sample of currently-active sex workers as trainees to become peer health educators. Recruitment posters and application forms were circulated to various local community-based organizations frequented by sex workers, local escort agencies, and on online fora that sex workers might use to find clients. Recruitment was modeled as a job application whereby candidates were requested to submit a completed application form, either in person at participating organisations or by email to the Project Coordinator (second author). Eighteen applications were received within the short advertizing period (1 month), which was better than the research team expected, given the nature of the position – i.e., the individual had to be willing to ‘come out’ as an active sex worker and be part of a research project that involved active peer engagement. The team short-listed nine applicants based on their answer to the questions “What brought you to apply for this position? What is your interest in being a Peer Health Educator?” During the interviews, the applicants were asked to describe their demonstrated leadership and networking skills, their access to social support to cope with any challenges that might arise during the life of the program, and whether their schedules fit the program’s requirements. Interviews were conducted, and positions were offered to five of the nine applicants (the maximum that the research budget could fund) who had the greatest leadership and networking skills, were available to undertake the training and had a strong social support system. Trainees varied in age, gender, sexual orientation, Indigenous background, socioeconomic status and sex work history. Their sex work locations ranged from independent indoor, webcam, escort agency to independent outdoor. Some worked full-time, others part-time and some were starting to transition out of sex work.

The training program was offered at the local sex workers organization and consisted of 16 2-h sessions, followed by 8 weeks of interactions in the community, during which participants took part in weekly 2-h debriefing sessions. Trainees received a cash honorarium of $40 at the end of each session. Snacks and beverages were also provided in order to increase comfort during the meeting. Sessions were offered by academic and community members of the research team, local service providers from sexual health clinics and HIV/AIDS service organizations and by community members with lived experience. Training sessions covered a range of topics of interest and concern for sex workers (see Table 1).

**Table 1:** Training Session Topics

· Empowerment Approaches to Sex Work	
· Becoming a Peer Educator	
· Honouring Diversity in Gender and Sexuality	
· Honouring Diversity in Indigenous Communities	
· Clients, Health and Safety	
· Health and Social Services Mapping Sexual Health for Sex Workers	
· Harm Reduction	
· Overdose Prevention and Naloxone Training	
· Meet and Greet with Health and Social Services	
· Practicums	
· Debriefing Sessions	

Data were collected from December 2015 to June 2016 through: 1) qualitative semi-structured interviews conducted by the first author with the participants prior to the training, after the training and at the end of the 8-week intervention phase (14 in-person interviews were conducted in total; one participant did not complete the third interview); 2) journals kept by the participants and Project Coordinator throughout the training program (5 in total); and 3) anonymous feedback forms collected from the participants by the Coordinator (second author) after each training and debriefing session (115 feedback forms).

The training program was offered at the local sex worker organization and consisted of 16 2-h sessions, followed by 8 weeks of interactions in the community during which participants took part in weekly 2-h debriefing sessions. Trainees received a cash honorarium of $40 at the end of each session they participated in. Each debriefing session was facilitated by the Project Coordinator and started with a check-in to see how everyone was doing. Trainees took turns sharing one of the interactions they had during that week, described how it went, what went well, what challenges they may have encountered, and whatever else they felt was relevant. The interaction was then discussed with the group so that everyone present could exchange tips on handling similar situations, share lessons learned, ask questions, and seek guidance and support as needed. During the 8 weeks of intervention, the trainees were asked to interact with four individuals each week, keep a journal of their interactions, and report back at the weekly debriefing session. At the end of each debriefing session, the trainees received $40 for their weekly interactions, as well as $40 for participating in the debriefing session. Additional files 1 and 2.

The semi-structured interviews were conducted in the lead author’s university office to provide a separate private space for the participants to reflect on the training program and subsequent intervention phase. The in-person interviews were audio-recorded and included 12 open-ended questions that varied slightly across the three-time periods to capture the progression of the training program and the changing perceptions and experiences of participants. These interviews ranged from 45 min to an hour. All three sources of information gathered from participants were transcribed and redacted to maintain confidentiality.

### Data analysis

Our thematic analysis of qualitative data followed Braun and Clarke’s [42] six phases: familiarize yourself with the full range of data, generate initial codes, search for themes among codes, review themes, define and name themes, and produce the final report. First, to begin working on a coding scheme, the second author organized the transcribed data into a single document, then extracted into a Microsoft Word document the excerpts from each interview related to four interview questions. The first three authors independently performed open-coding on these excerpts then met to discuss and eventually agree on a first draft of a coding framework.

**Fig. 1:**
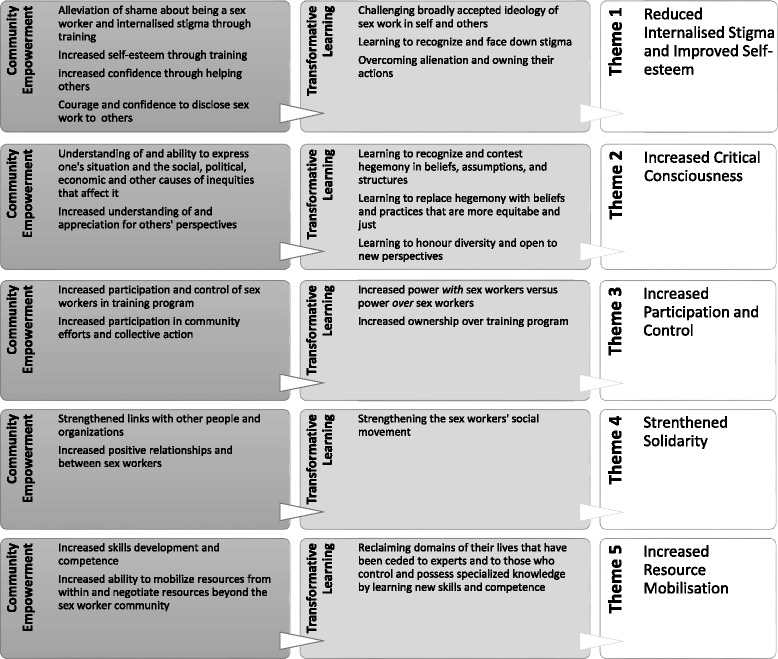
Broad Coding Framework

Next, the second author imported the data set into NVivo 10 software and applied the coding framework to the entire data, focusing primarily on condensing responses into smaller, descriptive units to reduce them into a more manageable form. She then produced a more refined coding strategy by searching for themes within the overarching transformative learning and community empowerment frameworks, focusing on the relationships between codes as well as relationships between larger themes (forming main themes and subthemes). Once the refined coding scheme was developed, the first and third authors independently coded the same set of data. The first three authors then compared the consistency of the separately-developed coding schemes, identifying the most salient themes and making minor modifications to the final coding framework based on discussions of discrepancies. The second author then applied this coding framework to the entire set of data, which the first and third author then double-checked to assure inter-rater reliability. A broad coding framework is offered in Fig. 1 to illustrate how each theme is positioned within community empowerment and transformative learning [43].

### Ethical considerations

Prior to data collection, the ethics review board at the University of Victoria approved the study. To ensure ongoing informed consent, participants were provided with the consent forms before each in-person interview. At each point, they had the opportunity to discuss the study, had their questions answered and were given the opportunity to withdraw their participation at any time without any detrimental effects. Participants were reminded throughout the training program that they had the right to share as much or as little information about themselves as they felt comfortable. They were also informed that if they had disturbing thoughts and would like help, they would be provided with a list of counseling services in the city. We attempted to minimize any inconvenience by creating a safe environment during meetings and made sure participants were well prepared to deliver peer education in the local community. Participants also provided input into this manuscript and a summary report, as did all members of the research team. To protect confidentiality, participants are identified as P1, P2, P3, P4 and P5 throughout the analysis.

## Results

**Fig. 2:**
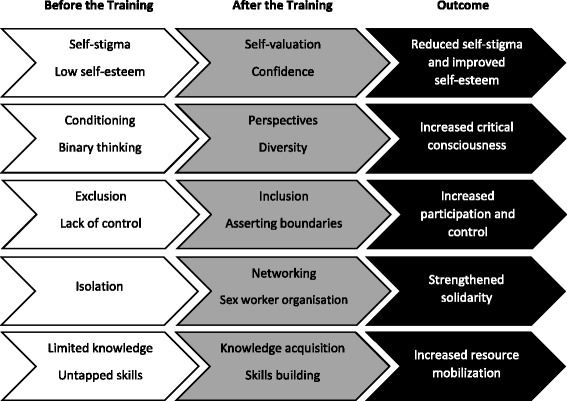
Community Empowerment and Transformative Learning of Participants in the Pilot Training Program

Figure 2 summarizes the five key themes comprising the community empowerment and individual transformative learning processes described by the participants.

### Theme 1: Reduced internalized stigma and improved self-esteem: *“That action doesn’t define me”*

Positive self-valuation and confidence are precursors to the development of empowerment [39]. Conversely, internalized stigma occurs when individuals’ adopt prejudice directed at a group they are part of, which often leads to lower self-esteem and lower self-efficacy [44].

Participants differed at the beginning of the program in how much sex work stigma affected their self-image. P2 spoke of a strong sense of self at the first interview, even when confronting negative judgment from health providers:

I was speaking with the doctor and told her that I was a sex worker, and she said, “Oh, so you’re getting out of that business, then, right?” that was her reply, and I said, “No, this is my job” and she just couldn’t comprehend it and was sort of upset…, treating me like I was just a wounded person and troubled and needed help. [I]nstantly I could see her glancing at my arm, seeing if I was an intravenous user and things like that, and that really disgusted me… I’ve never used heroin…I’m just your everyday girl that is in a different kind of business.

The sex work positive approach of the training program helped other participants challenge their own ideology about sex work and alleviate some of the shame they felt about their involvement in sex work: “Before I carried a lot of shame about being a sex worker. […] it ate me alive inside, some days. But you know what? Today it doesn’t. […] And it’s because of this project, that I can confidently say” [P5]. The training program helped this participant learn to recognize and face down stigma associated with sex work and come to terms with a sex worker identity. They learned to manage some of their internalized stigma:

I wasn’t…comfortable, even in my own skin as a sex worker. Participating in the project has allowed me to explore that, because that action doesn’t define me. And that’s one thing I’ve learned, is because there’s such a stigma about sex workers and that’s why I was never, ever comfortable. [P5]

Others noted increases in their self-esteem through the program: “I think it really helped me with… more self-esteem” [P1]. An interaction between P2 and a family member about being a participant resulted in reflection on some of the intangible gains from being part of the program: “I help my peer[s] and…it builds my confidence and…I feel a lot of [happiness] giving back.” [P2 Journal]. Reduced internalized stigma and increased self-esteem also provided participants with the courage to overcome alienation, own their actions, and disclose their sex work to their loved ones:

Since I’ve been participating in this initiative, it’s allowed me to be more honest with [my partner]… If it wasn’t for this program, I don’t think I would have been as forthcoming… It was always my dark, dirty secret. You know? [P5]

The transformation that led to this reduced internalised stigma and increased self-esteem expanded to an increase in participants’ critical consciousness, as described in the next section.

### Theme 2: Increased critical consciousness: *“Divide the black and white and open that gray area”*

Critical consciousness is developing the capacity to challenge ideologies, values, beliefs, myths, explanations, and justifications embedded in language, social habits, and cultural forms and is an important goal in enhancing community empowerment [17]. Through critical consciousness, adults learn to recognize and contest hegemony in these beliefs, assumptions and structures [41]. The participants expressed that they came to realize how their ideologies had been conditioned by society: “Learning about myself and how I’ve been “brainwashed by society”” [Anonymous Feedback Form].

Critical consciousness was particularly raised around issues of diversity, either by participants who became conscious of their own limited views or by participants challenging others’ views about aspects of their person, including their cultural background and sexual orientation:

The biggest challenge for me were people would try and label my [sexual orientation] identity… The biggest challenge was exposing, and I learned it in this training, exposing their binary thinking… So my biggest challenge was to divide the black and white and open that gray area. [P5]

Participants learned to honour more diversity, expressed becoming more empathetic and open to others’ perspectives, and more sensitive to language used to describe others: “I’ll say empathy. Being empathetic… towards other people” [P1]; “What could be improved: Older language such as “transsexual”” [Anonymous Feedback Form].

Participants also spoke about learning about diversity from the various speakers during the training sessions, as well as from each other: “The expanded expression of terms of gender and sexuality were very enlightening” [Anonymous Feedback form].

Participants also came to realize that sex workers had various perspectives about sex work:

I like this job so much and I love all the opportunities that it gives me, I assume that everybody feels the same way. So, it was interesting. I expected to go into a really sex work-positive space. And it wasn’t really like that. And that’s not a terrible thing. It is good to see the other…side of the coin– yeah, the diversity of it. [P4]

Through increased critical consciousness, participants grasped how stigma against sex workers can be perpetuated, either by the presenters during the training sessions or by organizations that serve sex workers. Despite claims of a sex work positive approach, they contested this hegemony by pointing out that there was a focus on street-involved sex workers in some of the training sessions:

I felt there was a bit of a “pro-exit” undercurrent to the presentation. Seemed to focus a lot on how awful the experiences of… sex workers have been and not a lot of focus on how we or any other group can help make positive changes… I’m being kind of critical of specifically some of the elements of it… a lot of the stuff… it’s focused on, like, heroin overdoses and homelessness and stuff… Sex workers are the people who are all around you. They’re working in a coffee shop, and they’re going to university, and they’re walking their dog… Like really, really normalizing us. But I haven’t necessarily always seen that in the material that’s being presented. [P4]

During the training program, participants interacted with representatives from various health and social services from the area. They engaged in discourses around sex workers and people who use drugs. They challenged stigma and saw this as helping others, normalizing sex work, and bettering society:

P2: I was open about being a sex worker and I had this doctor tell me “Oh, you know you can do another job. You don’t have to do this.” And, you know, “You’re a bright [person]” and all this stuff. And I’m like “I know that about myself… I’ve chosen to do this. I have other opportunities, but this is what I choose to do”…

Interviewer: So the education helped you point that out to people in the hospital?

P2: Yeah, and… have the strength to make a point of it… and that it would better society and help other people, it was really good. [P2]

Two training sessions provided an opportunity for participants to provide feedback to the service providers about their experiences with the services: “Since the [community health centre] nurses have come to the class. And since we educated them on how it comes from our perspective… they’ve really stepped up and been understanding” [P2]. “Service providers were great about receiving feedback” [Anonymous Feedback Form]. The discussion also left participants with strategies on how to handle situations of stigma with service providers or other community members.

Critical consciousness about the experiences of others within their networks was also discussed, and some advocates acknowledged how they do not always consider others’ perspective, especially that of their sex industry clients. Having the opportunity to learn about the perspectives of these other groups was enlightening for some: “This was very interesting. Helped open the door to clients, that there is more to them than acquiring a service” [Anonymous Feedback Form].

The development of critical consciousness was only one aspect of transformative learning and community empowerment that emerged from participation in the training program. There were also signs of improved sex worker participation and control over the training program, as described in the next section.

### Theme 3: Increased participation and control: *“Far from that teacher approach”*

Community empowerment-based responses prioritise the engagement of members of the population of interest, who have historically been excluded in the development and delivery of programs [13]. Improving sex worker participation and sharing control over program management was an important part of this program and was built into the participatory study design. Participation included having sex workers on the research team, engaging the participants in the development and content of the training program, and integrating their input into reporting on the results.

In an effort to shift the power dynamic from one of power *over* sex workers to power *with* sex workers, approaches and conditions were used to foster sex worker participation. Frequent reminders that this training program was also *their* program to develop and shape led to increased control by participants. First and foremost, the teacher-student dynamic was eliminated and replaced with a more horizontal power dynamic, which was noticed:

I felt like it would be more like teacher and student type thing. And then… everybody in the group, you know, we were just like a group of friends… we were all working our own little ways on different stuff we had about ourselves to helping other people and all those things, and then at each week coming in and sharing experiences; it was a lot better. And then also, with [the Project Coordinator], you know she was far from like that teacher approach. And it was just so relaxed and fun. And I found we all could share, and let down, and actually learn more… And learn a lot from each other. [P2]

The session facilitator and presenters remained open to input from participants: “The facilitator’s openness to changing our curriculum was inspiring” [Anonymous Feedback Form]. For example, for the two sessions on sexual health, participants asked that one be focused on the transmission of STBBIs in the context of sex work and the other on their own personal sexuality outside of sex work. Participants provided feedback throughout the sessions, which were integrated into the curriculum, experimented with leading the discussions during the debriefing sessions and provided their expertise to some of the presenters: “I felt the need to raise the awareness… Training the trainer” [P5]; “I felt like I chaired this meeting” [Anonymous Feedback Form].

In time, participants increasingly took ownership of the training program, including changing the title from Peer Health Educators, given at the start of the program, to Peer Health Advocates, a title P5 said better reflected the role: “That’s one thing that I wanted to share too, is even in our little intimate circle, how it evolved. How we were able to, you know, strategically and proactively even change the title of our training” [P5]. As advocates rather than educators, participants were “being viewed as a resource” [P5] and that: “we don’t always have to have the answers but we can always refer them to somebody that can give them the answers” [P5]. On one occasion, one of them intentionally arrived early to suggest rearranging the room format so that all attending sit in a round table setting. This seemed to work well for the group, as expressed in the feedback form: “The round table change brought a more group setting, eliminated cross talk, removed the teacher student atmosphere” [Anonymous Feedback Form]. This set up was retained for the rest of the training sessions.

Participants increasingly demonstrated initiative in contributing to the development of the training program and experimenting with the implementation of the intervention. Examples of this included asking others outside the program for feedback, taking actions in their circles to improve access to safer sex and drug use supplies and online interactions with sex workers:

I’ve started running a blog that’s a support for mentally disabled sex workers. I was following it for a while, and then because of the education portion of it [training program] I was like, “Hey, maybe I can help out”. [P3]

Increased control was expressed strongly by participants asserting their boundaries as a result of the training. While most participants had been strict about safer sex practices in sex work all along, such as P3: “I’ve had multiple clients who…have used various tactics with me to try and get around using barriers. All of them I just walked out on. It’s actually non-negotiable”, others relayed difficulty asserting their boundaries around safer sex practices both in sex work and in personal life. These individuals described acquiring greater confidence to assert themselves with respect to safer sex practices through the training. As P1 noted: “I think there is more respect between my clients and me now. Like you know, just because I’ve been really strict about, you know, using safe sex practices and so they don’t question it like they did before” [P1]. Asserting boundaries with regard to their role during the intervention and making choices about how far one would go to help others also developed during the training. During debriefing sessions, one participant reported being aware of not respecting their own boundaries by housing someone for the night. Another participant was asked to coach someone’s partner on how to get into sex work. The participant declined the request. Yet another participant stated that:

A [sex worker friend] is on methadone and on the same meds as I am… She told me she relapsed and asked me if she could have some of my urine. I didn’t want to enable her but I didn’t want her to suffer the huge consequences. In the end, I didn’t give it to her**.** [P2]

In sum, by being involved in this research project and having the opportunity to assert one’s thoughts and opinions on the program, some participants noted feeling positive about “… getting my voice heard” [P3] by the researchers and service providers. Participants were also able to extend their voice to peers and others in their communities through the research program, which led to critical networking opportunities and strengthened solidarity.

### Theme 4: Strengthened solidarity: *“It’s like family out there”*

The training program fostered group empowerment by strengthening relationships and building solidarity among participants, as well as with the local sex worker organization and with other sex workers in the community. This strengthened solidarity contributed to strengthening the sex workers’ social movement by expanding relationships between sex workers and the sex worker agency. The support provided by the local sex worker organization was important in empowering participants to identify as sex workers: “I would never, ever, before being associated with [local sex work agency] be able to identify as a… sex trade worker. It’s very liberating to have all of that come forward and know that there’s support services in place” [P5].

Bonds formed among participants during the training and extended beyond the weekly sessions. These positive relationships mitigated isolation brought on by (internalized) stigma. It provided an opportunity for the group to work as a team, network with each other, support each other, and become friends:

I found sanctuary within this program to share, to view other workers as my equals. Whereas I often felt judged, even by other sex workers… Because ideally if I isolated, it kept my secret stronger. I was the stronghold to my own secret. So I would never divulge or disclose on a level that I did within this program, and it created friendships. [P5]

Participants expressed a desire to stay in touch beyond this project: “Our little group has sort of all connected and we’ve had each other’s phone numbers and we can text each other if there’s a problem with something and there’s a real support in that” [P1].

Community empowerment among sex workers extended beyond the group of participants. One participant expressed how telling other sex workers about their involvement in this training had resulted in opportunities for knowledge sharing: “I found a lot of them are sharing more… I put it out there, at first, it’s [peer health advocate program] something I talked about… And some have shared some personal, really personal things and stuff I can help them with” [P2]. Another participant stated: “the social network that I have of… sex workers was actually minimal. And participating in this [training program] expanded that*”* [P5]. Participants expressed a desire for ongoing training so that more sex workers could become involved, as well as more networking between sex workers through ongoing group meetings:

I think it would be good if we could get like a bunch of us trained and out there… if you’re on the street and you’ve got this training, you can help so many people with what you do… Because… in town here with sex workers and people on the street, it’s like family out there and everyone is connected to someone. [P1]

Realising the commonalities between sex workers and having the support of peers and allies helped build cohesion and group empowerment:

I just feel that we’ve got something in common; being sex workers… you might be nervous at first to even bring it up. But once you get talking, everyone goes to the same thing. Similar situations, and problems, and worries about things. [P1]

Group empowerment was also expressed through solidarity between sex workers. Participants stood up for peers, accompanied them to a safe place, provided safer sex supplies and linked them to services:

The girl had had a client take the condom off and when she was saying, “Stop” he wasn’t stopping and… when she finally kicked him off and had him come downstairs, she was really horrified and felt violated, understandably […] I took the rest of my shift off, took her to the police. [P2]

A lot of these women that work are homeless… And there was this girl on the street one night crying her eyes out because her boyfriend had just dumped her downtown… She was a young girl and I was really worried about her safety because she wasn’t with it at all… she was cold…we walked together… I said, “Please go there, you can get a coat”. [P1]

Participants also expressed the need for more resources for people who are new to the sex industry, to better educate them about what to expect:

I think that there needs to be a lot more education for people coming into the business as, basically, as escort training. Safety, forewarnings, like, clients love new girls because they’re a fresh face but mostly because they’re vulnerable and new and they don’t know all the tricks, they don’t know all the rules*.* [P2]

Finally, access to new knowledge and resources, and skill development helped participants mobilise resources in the sex worker community.

### Theme 5: Increased resource mobilisation: *“It helped me realize more people I can help”*

Mobilising resources within the sex worker community started with skills building and knowledge acquisition, an important area of growth and empowerment for participants. Learning new skills and gaining new competence contributed to sex workers reclaiming domains of their lives that were controlled by experts who possessed specialized knowledge, thereby helping to shift some power back into sex workers’ hands. Increased resource mobilisation involved learning communications skills, active listening, and strategies to approach others with more ease, at times resulting in changed practices in sexual health:

I’ve always been getting my testing frequently. I do it every 2 months. But, I’ve been doing more of that and checking for other things that I didn’t usually check for… but learning that there’s sometimes stuff you can’t see and… you just don’t know; they don’t have pain or whatever. So I’ve been more thorough about that and more thorough about my blood work. [P2]

Participants learned about available health and social services in the area and others’ experiences with the services. In addition to being able to use this information to help others in the community, they were able to seek help for themselves, including safer sex practices in sex work: “This project has helped me identify where and when I can get help” [P5]; “I actually hadn’t been to [the local sex workers organization] before this training, so it was directly because of the training” [P3]. Participants were also better equipped to negotiate safer sex practices with clients:

It made a difference in the way of being more open about problems with [clients]… just being able to get it out there about… how I felt about… using safe sex practices. And with them, I’m more comfortable in that way. [P1].

More generally, participants had greater confidence discussing sexual health: “I have, I think, more confidence knowing, like, a lot more education I have and a lot more resources that I know about” [P2]. They had gained competence in speaking out on these issues and felt more approachable to others:

[The training] has really helped me put it into words and express what I want to… I couldn’t articulate well enough to come across as a nurturing, inspiring person. I believe I’m more approachable because of the training to somebody who wants information. [P5].

Participants also related being more confident venturing into the community and helping others due to the training and the knowledge gained about available resources. Participants linked those they came in contact with to health and social services through referrals, accompaniment and follow-up and connected sex workers with a peer-led sex workers’ group, health clinics, sexual health clinics, and other services.

I told her where to go… She was actually from [out of town]. I actually got her to go into… the clinic there. And I set [her up with a doctor]; he’s really good there. He works with people that work… And now she got an appointment in just to get herself checked out, like bloodwork and everything. Because she’d been taking some risks, right? [P1]

Another commented:

A sex worker friend of mine told me that she needed a new IUD inserted. I was able to direct her to [the sexual health clinic], and give her one of their pamphlets. I was able to offer help that I didn't know existed before we did our training. [P4]

Participants also used their training to discuss sexual health with clients, refer them to services as needed and encourage testing for STBBIs.

I had an older client tell me “Never been tested before.” I was horrified to hear that… So there’s been a lot of education to really older clients. So yeah, I’ve, you know, said “Hey,” you know, “Check this place out. It’s really, you know,” and always: “If my wife ever found out…” and stuff. But I explain, you know, “it could really affect your health” [P2].

Beyond health services, participants were able to play a role in connecting their peers with free services or services that could improve their financial or housing situation.

She wasn’t really aware of where to get free condoms and stuff, which was surprising to me. But now she’s going there regularly… and I ran into her… and she goes “Oh, I’m so glad for your [suggestion]” because she goes “I usually would buy those and it would cost me”… So she’s been going in there and getting everything for free, and she’s just thrilled about that. [P1]

Without the project I wouldn’t have learned about all the subsidies out there; I wouldn’t have learned how to investigate resources. So it was really helpful… I didn’t know the government gave our province money for housing… So participating in this initiative has given me the courage to ask those questions and say, “Okay, well how can I get more assistance here?” [P5]

With the knowledge gained about available health and social services for sex workers in the area, participants were able to refer many people in the community to various services, thereby opening a lot of doors to services to meet needs: “I’m on the up and up… on where to go in town” [P1]. Participants noted that information sharing and connecting with others encouraged sex workers to return for help:

If something else comes up in their mind that they need help with, they’ll come back to me… it could be drug use, or like, talking to a drug counselor because they’re thinking about getting clean. Where to go for some housing. [P1].

Participants were also able to contribute to resource mobilisation in the community by doing secondary distribution of safer sex and drug use supplies:

I do have interactions that are from all over the training. Harm reduction supplies is one. Like actually, I specifically got a nice large handbag and filled it with harm reduction supplies. [P3].

I always keep supplies on me. Like I actually distribute rigs for [a community organization], even though I’m not really connected… But quite often when I’m down there, I’ll grab supplies for people. Like I can get bags with everything in it, and I’m just handing them out on the street. [P1].

Some participants felt rewarded by opportunities to help others: “It’s giving back to the community, which is really big for me” [P3]. Another put it this way:

And it made me think about how important it is to try and prevent things like [STBBIs] in the sex trade… if I can increase my own…level of understanding of STIs and if I can help spread that in the community then that’s good for everybody… Because it’s just about helping people. [P4]

Participants specifically learned new ways of helping others through the debriefing sessions during the 8-week intervention phase of the project: “Hearing everyone’s debrief was helpful. It helped me realise more people I can help” [Anonymous Feedback Form]. After the project ended, participants continued to give back to the community by volunteering, found employment in street outreach, or were planning on continuing to interact with people in the community:

Being active at [local sex workers organization] as a client during the week and then volunteering there… Something I wouldn’t ever thought of doing before, but they were very helpful… And it’s the only way I could think of giving back. [P5]

My biggest goal actually right now is to… connect one person a week to adequate resources, so be it mental health, so be it the [downtown clinic], so be it addiction services… that would help them along the way, I would feel like I gained from this project. And that’s not an unrealistic goal. [P5]

I think that even if I don’t secure a job, you know, a paid job doing it, I’ll still be out there doing what I was doing when I was taking the course… I do enjoy doing it and helping people. [P1]

In summary, the findings reveal that participants gained improvement in self-esteem and reduced internalised stigma, increased their critical consciousness, benefited from participation and control over the training program, which also translated into more control and asserting boundaries related to outreach work and sex work, strengthened solidarity with other sex workers, and contributed to increased resource mobilisation in the community as a whole.

## Discussion

This pilot health education program, designed with and for sex workers, aimed to enhance community empowerment and transformative learning to better enable sex workers to shape personal practices around health promotion and prevention strategies, and to contribute to improving access to health and social services within the local community. Trial program activities were carried out with the sex worker community taking on an active role within an enabling environment in collaboration with various partners. Sex worker representatives had already been collaborating with researchers and community partners for some time. The idea for this project emerged from these collaborations. Findings reveal successes in beginning to enhance community empowerment through the pilot program, as well as evidence of transformative learning among participants. This research adds valuable information to the literature on interventions that aim to enhance community empowerment and transformative learning processes.

First, the continuum of community empowerment begins with personal empowerment [37]. Our findings of reduced internalised stigma and improved self-esteem through participation in the pilot program, whereby participants reported greater self-confidence and belief in themselves, bode well as catalysts to developing a sense of control and power, thereby launching a process of personal empowerment. One of the more powerful effects of the program’s sex work positive approach on personal empowerment was the alleviation of shame and stigma associated with being involved in sex work, which led some participants to be more confident and better value themselves as a worthy person who does sex work for a living [18].

Bringing together a group of sex workers challenged the stigma that often deters stigmatized groups from meeting together [40]. Our pilot program targeted intrapersonal dynamics to reduce stigma expression and the impact of stigma on sex workers [44]. It brought together a small group of individuals who share sex work-related stigma but vary in terms of other stigmas associated with poverty, sexual orientation and gender identity, age, and ethno-cultural background [45]. The pilot program combined both education-based strategies, whereby information was offered to challenge sex work stereotypes, and contact-based strategies, where there was face-to-face exchange between sex workers and others [44] in an attempt to challenge and reduce stigma expression and better integrate sex workers into the community. Participants came to recognize how their views about sex work are influenced by broadly accepted societal ideology of sex work, and were able to face down that stigma, overcome alienation to a certain extent, and own their choice of work [41]. Our findings suggest that the sex work positive approach to the program helped mitigate the internalized stigma some participants felts about sex work involvement. The program encouraged instances of challenging stigma expressed by health professionals and training presenters. The aim of this multilevel approach to addressing stigma was to create favourable conditions for long-lasting effects. The impact of the intervention on sex work stigma was limited by its pilot, non-sustained nature, though showed promising potential to address stigma in participants as well as among service providers. This shift provided a fertile ground for increased critical consciousness or critical awareness.

Still at the individual level of personal empowerment [37, 38], critical consciousness or critical awareness has been described as a process toward autonomy whereby one comes to understand and is able to express one’s situation and the factors that affect it, such as the social and political contexts and inequities [18, 38, 46]. Through this critical awareness, adults learn to break apart prevailing sets of values and beliefs, unmask power that leads to inequity and oppression, challenge such ideology and contest its resulting hegemony [41]. In our pilot program, participants came to see how their worldview was shaped by conservative societal values, learned to challenge personal views and those of others, and became more open and empathetic to a wider spectrum of identities related to gender, sexual orientation and the perspectives of others (e.g. clients) [41]. Participants heard various perspectives and experiences with sex work and expanded their views beyond personal experiences. In our consultation with participants about this manuscript, participants confirmed that this greater critical consciousness had changed how they interact with people of various genders and sexual orientation, suggesting that they had adopted more equitable and just practices in their everyday lives [41].

Participants challenged stigma against sex workers in the community by fighting alienating forces [41] and speaking up when faced with derogatory comments or negative assumptions about sex workers. Some participants also showed increased ability to critically reflect on and choose a healthy course of action in terms of asserting boundaries around safer sex practices, showing promise that such a training program could lead to better STBBI prevention among sex workers. These displays of critical consciousness paved the way for increased participation and control over the pilot training program.

Our program enhanced sex worker community empowerment through meaningful participation in and control over the design and implementation of the pilot program [25]. Our dialogical educational approach to the program, with a specific intention of shifting the power relations away from a teacher-student relationship to one of power *with* sex workers, reflected democratic practices that foster transformative learning [41]. This approach was successful in fostering critical thinking, agency and solidarity among participants [25, 47]. Sex worker group empowerment surfaced in the community empowerment continuum [37] as participants’ control over the program increased with time and progression of the training sessions. Participants also said they experienced less judgment from health care practitioners after speaking out about stigmatizing treatment in healthcare settings through the training sessions, thereby contributing to reducing barriers to access that sex workers face when accessing health care in the local community.

Signs of participants getting more involved and shifting their mindset toward one of more ownership over the program were documented, as they realised that their input was integrated into the program [2]. Brookfield [41] refers to this process as ‘reclaiming reason’, whereby participants retrieved domains of their lives that had been ceded to academic and service provision experts, and integrated their own specialised knowledge into the program. Changing their title from Peer Health Educators to Peer Health Advocates was the penultimate example of control and ownership of the program. Having clearly defined roles and responsibilities and reiterating to the participants that they played a key role in the program’s development and implementation contributed to this increase in participation and control [17]. Participants also had the opportunity to exercise control over improving services for sex workers by providing local service providers feedback on their experiences with the services and witnessing changes as a result. The program thereby contributed to community mobilisation by resulting in some changes to services and community contexts that support sex workers [47].

Sex worker empowerment grew as positive relationships and strengthened solidarity formed amongst study participants and beyond [16, 17, 40]. This social movement contributed to encouraging sex workers to collectively organise and take part in improving conditions for sex workers [41]. In the bigger picture, these findings reveal that the pilot training program shows promise in sex workers reclaiming some power over domains of their lives that have been ceded to those in control in the current societal and political context. Participants became known to other sex workers in the community and sought after for knowledge and assistance. Participants also expressed a desire for a sustainable program so that more sex workers could take part in and benefit from it. The need for health information and care for those new to the sex industry was emphasised. Holding the training sessions at the local sex worker organization was instrumental in providing a safe space and empowering participants to positively identify as sex workers. Our pilot program also contributed to reducing sex worker vulnerability to STBBIs and enhancing access to health and social services by increasing a sense of shared identity and networking among sex workers, and by touching on subjects beyond sexual health, such as stigma and discrimination [25].

Finally, increased resource mobilisation through skills building and knowledge acquisition [18] contributed to behavioural change. Peer education and community empowerment programs have been shown to be effective at improving behavioural outcomes related to STBBI prevention [25, 32, 47]. In our pilot program, participants were more informed and confident about discussing sexual health in general, and negotiating safer sex practices both in work and personal relationships. They also expanded access to health and social services by informing and providing information about services to their peers, and at times accompanying them to access services [17]. In our meeting about this manuscript, two of the participants reported having since used their naloxone kit obtained during the training to save someone from an opiate overdose. Participants’ own life conditions improved as a result of participation in the program by being able to access health and social services that were previously unfamiliar or unknown. These findings once again suggest that such a training program has the potential to increase the effectiveness of STBBI prevention and treatment. They also reveal more situations in which sex workers reclaim power, through skills building and knowledge acquisition, over domains of their lives that have largely been controlled by health and social services professionals [41].

Community mobilisation, where we sought to generate change beyond the sex worker community to help create a health enabling social environment where the program took place [47], was somewhat successful, though limited by available resources and time. Supportive partnerships exist with local health and social services, though the project did not extend to actors and agencies who have the political and economic power to effect structural change. The findings from this study will assist sex workers and partner organizations with advocating for structural changes. The pilot program did provide a transformative space for critical dialogue [47] between sex workers and health and social services representatives, and led to some changes in service delivery.

Contextual factors played a critical role in the program’s success. The symbolic context of meanings, ideologies and worldviews provided through the program, namely a sex positive, sex work positive, harm reduction, human rights approach, conveyed respect and helped some participants reframe personal views of sex work, while others received affirmation for an existing positive view [47]. It also provided participants with the tools and confidence to challenge stigma against sex workers when encountered in the community. To help with the material context of poverty faced by most participants [47], the program provided some vocational training for outreach work [25], remunerated participants, and provided reference letters to participants at the end of the training. One participant was subsequently hired as a peer outreach worker by a local agency. These measures therefore had some impact on improving the participants’ material needs. Finally, in terms of the relational context [47], sex worker leadership and power over decision-making was fostered in the program in an effort to shift traditional hierarchical power relations to a more horizontal plane [41]. Future programs, with full ownership, delivery and implementation by sex workers in partnership with community organizations would likely further improve the relational context. Further adaptation of a sustainable program, and enhanced involvement of sex workers in the delivery and implementation of the program, would likely enhance solidarity between sex workers as well as community mobilisation. Peer education approaches have been especially successful in affluent countries, in contexts where sex workers already had strong agency, solidarity, a common identity and a degree of community mobilization [47]. Our success is likely attributable, to a certain extent, to the fact that the program was situated in the local sex workers’ organization, in a city where the greater community had been mobilising around improving services and conditions for sex workers and our findings support that.

With regard to sustainability [2], which usually comes through broader social engagement, community acceptance and a focus on a long-term sustainable intervention, this pilot project planted some seeds in what appears to be fertile ground in one particular city of Canada. Participants suggested that more focus on safety and anti-violence (e.g. self-defence training) in future programs might enhance the capacity to obtain funding while addressing sex workers’ increased exposure to violence as a result of changes in the law. There were indications that there was sex worker community will and community partner capacity to continue this project beyond this pilot phase. The sex worker community was receptive to the role of Peer Health Advocates. This program served as a proof of concept, with promising results, which will feed into pursuing resources to sustain it. The strengthened links to health and social services, both through their involvement in the training program and their interactions with Peer Health Advocates, also bodes well for focusing on a long-term sustainable intervention in the near future, if funding is secured for further study.

Our study is not without limitations. The small number of participants limited the generalisability of the findings. Recruitment of participants strove for diversity along Indigeneity, gender, age and work location, although without doubt many perspectives were missed. Recruitment was also approached as a job application for those interested in promoting health amongst sex workers. This approach may have deterred some candidates from applying though did support our goal to recruit natural leaders in the community. An alternative approach would be to recruit interested participants through one or more of the ongoing programs run by the sex worker organisation that include Drop-In Centre/Wellness Clinic, Housing and Community Support, Small Business Training Program and Indoor Workers Dinner and Education Group.

The pilot program’s short duration of less than 7 months also limits generalisability of the results relating to transformative learning and emerging community empowerment among participants. Further interventions and studies of a longer duration could build on this initiative and determine whether this approach could lead to full ownership of the training program by sex workers and sustainability through the local sex worker organization.

The pilot program was also implemented in an urban context where there has already been concerted efforts by various local actors (i.e. community organizations, police) to improve the well-being of sex workers. However, urban contexts vary significantly across Canada in regard. It is unlikely that the community empowerment approach outlined will be successful in settings that have not developed an environment were sex workers are viewed as active members of the community and deserving non-judgmental health care and other services, similar to other citizens.

Data were only collected through observations and interviews with program participants, given the limited time and resources. It would be insightful to obtain the perspectives of the community partners and researchers involved in the project to determine if they experienced a transformation by taking part in a project on sex worker community empowerment. Future studies should include a larger number of participants, quantitative measures of key concepts (internalised stigma, self, esteem, etc.) and also collect data on perceived stigma and discriminatory behaviours toward sex-workers among community-members to enable a more comprehensive understanding of the benefits and challenges of a community empowerment framework and whether a transformative learning approach will in the long run improve sex workers’ access to health services and enhance their health equity on other fronts.

It would have also been great to have been able to assess the impact of increased community empowerment and transformative learning obtained through this training program on the effectiveness of health prevention and treatment services for sex workers. However, this study was a pilot done with a small catalyst grant and such an assessment lied beyond its scope. The glimpses of a positive impact on STBBI prevention and treatment provided in our findings are nevertheless promising.

## Conclusion

This study was a qualitative assessment of sex worker community empowerment through a trial peer health program. The program reduced sex workers’ internalised stigma and improved their self-esteem, critical consciousness, participation and control over the program and their life conditions and solidarity with their peers, as well as their knowledge of and access to health care. The participants were more informed and confident about discussing sexual health in general, and negotiating safer sex practices, both in work and personal relationships. Access to health and social services also expanded by informing and providing information about services to the participants’ colleagues, and at times accompanying them to access services [17]. These outcomes are critically important among populations such as sex workers who, due to the combined effects of stigma and criminalisation, face significant barriers to accessing basic pertinent health knowledge, quality health care, and other public resources [48].

This pilot study was, to our knowledge, the first of its kind in Canada. The findings reveal that the approach proved successful in enhancing sex worker community empowerment and transformative learning in one urban setting. This promising proof of concept built the foundation for a long-term initiative in this setting. Other jurisdictions in Canada that have made some inroads in creating a welcoming and respectful local environment for sex workers could learn from the initiative and perhaps adapt similar programs in their area.

## Additional files

Additional file 1: Pre-Training Interview with Peer Health Educators: Open Ended Questions (Interviewer Copy). (DOCX 43 kb)

Additional file 2: Peer Health Educators Training Program Session Evaluation Form. (DOCX 12 kb)
