# Supplementary material for: Sex workers as peer health advocates: community empowerment and transformative learning through a Canadian pilot program
Source: Int J Equity Health. 2017 Aug 30;16:160. doi: 10.1186/s12939-017-0655-2 (PMC5577770; doi:10.1186/s12939-017-0655-2)
Supplement: Supplementary file 1 — Pre-Training Interview with Peer Health Educators: Open Ended Questions (Interviewer Copy). (DOCX 43 kb) [file 12939_2017_655_MOESM1_ESM.docx]

**Pre-Training Interview with Peer Health Educators:**

**Open Ended Questions**

**Interviewer Copy**

*In this pre-training interview I would like to explore in greater detail many of the topics we will be covering during the training. The purpose of these questions is to gain a more complete picture of where you are at in terms of sexual health practices in the sex industry at the beginning of the project..*

**1. Can you tell me about how you first became involved in sex work and what your experience has been overall?**

[Interviewer probes: What led to the decision to enter the sex industry? What else was going on in your life to help you make this decision?]

**2. How does sex work compare to the other kinds of work you have done?**

[Interviewer probes: What keep you in sex work? What are the things about sex work that you find good compared to other jobs you have had? What are the bad things compared to other jobs you have had?]

**3. Can you tell me about the things that you currently do to take care of your health as part of your sex work? What types of things do you do to practice safer sex? What about safer drug use? For example, can you walk me through the typical things you do before and while on a date to take care of your health?**

[Interviewer probes: For example, what is the screening process like for your clients? Do you ground rules with your clients? If so, what are they? Are there things you wished you did more often, or resources you wish you had at your disposal to take good care of your health? Are there things you do not do for safer sex or safer drug use? Can you tell me how you make those decisions?]

**4. Have you accessed health care services for work-related health concerns? Do you feel that you have access to the health care you need?**

[Interviewer probes: Can you tell me more about the quality of care you received? What worked

and didn’t work for you within those interactions? Do you feel you have access to information about how best to take care of your health in your work? About safer sex practices and safer drug use practices? How important is the atmosphere of the health clinic or office, the personalities of the staff, or the level of confidentiality that is you receive, among other things? Do you ever feel stigmatized in certain clinics or offices and if so, what is it that leaves you feeling that way? What would be an ideal health encounter for you? Do you know about the various health services in Victoria where you could get this information?]

**5. Do you ever talk about safer sex practices with other sex workers? What sort of things do you discuss with each other?**

[Interview probes: Are there good times to have discussions with other sex workers about these things? How do you exchange information? Where do you find information and supplies to help you take care of your health in your work?

**6. Do you ever have disagreements with your clients about safer sex practices or safer drug use practices?**

[Interviewer probes: What makes a good client versus a not so good client? What are the factors that you feel leads to tension or conflict in your interactions with clients? What do you do in these circumstances? Or how do you avoid these kinds of circumstances? What do you wish you knew about how to handle these discussions?]

**7. Do you ever talk about work-related health concerns and practices with your clients? What sorts of things have you discussed with your clients about work-related health practices?**

[Interview probes: Do you have any practices that you insist on in your work with your clients? Do you find it easy/difficult to talk to them about it? Do you know where to get supplies? Are there things you wish you knew to make this discussion easier?]

**8. Do you ever talk about work-related health concerns and practices (e.g. safer sex practices, safer drug use practices) with managers or bosses of your sex work? What sorts of things have you discussed with managers or bosses about work-related health practices?**

[Interview probes: Does your manager or boss have practices that they insist on in your work? Do you find it easy/difficult to talk to them about it? Do they provide you with supplies for you to better be able to practice safer sex and safer drug use? Are there things you wish you knew to make this discussion easier?]

**9. Do you ever talk about work-related health concerns and practices with your romantic partners? What sorts of things have you discussed with your clients about work-related health practices?**

[Interview probes: Do you have practices that they insist on in your intimate relationships? What about your partner(s)? Do you find it easy/difficult to talk to them about it? Are there things you wish you knew to make this discussion easier?]

**10. Can you tell me what brought you to want to be a Peer Health Educator?**

[Interviewer probes: What led to the decision to apply for the position? What do you hope to get out of the training and being involved in this research project? What skills do you hope to develop through this project?]

**11. Do you have any concerns about becoming a Peer Health Educator?**

[Interviewer probes: Do you have any fears about the training itself? The research aspect of it?]

**12. Is there anything else you would like to add about how you think this Peer Health Educators training program will assist you in taking care of your health in your work and help your peers take care of their health as well?**

*This is the end of the interview. Thank you for taking the time to speak with me. It is much appreciated!*
