# Supplementary material for: Sex workers as peer health advocates: community empowerment and transformative learning through a Canadian pilot program
Source: Int J Equity Health. 2017 Aug 30;16:160. doi: 10.1186/s12939-017-0655-2 (PMC5577770; doi:10.1186/s12939-017-0655-2)
Supplement: Supplementary file 2 — Peer Health Educators Training Program Session Evaluation Form. (DOCX 12 kb) [file 12939_2017_655_MOESM2_ESM.docx]

**Peer Health Educators Training Program**

**Session Evaluation Form**

**Session 1: Meet and Greet and Orientation – December 14^th^, 2015**

1. What worked well for you with this session?
2. What could be improved with this session?
3. Other comments?
